# Team communication amongst clinical teachers in a formal meeting of post graduate medical training

**DOI:** 10.1007/s10459-015-9627-8

**Published:** 2015-07-31

**Authors:** Irene A. Slootweg, Albert Scherpbier, Renée van der Leeuw, Maas Jan Heineman, Cees van der Vleuten, Kiki M. J. M. H. Lombarts

**Affiliations:** Performance Research Group, Center of Evidence-based Education, Academic Medical Center, University of Amsterdam, PO Box 22660, Amsterdam, The Netherlands; Department of Educational Development and Research, University of Maastricht, Maastricht, The Netherlands; Faculty of Health, Medicine and Life Sciences, University of Maastricht, Maastricht, The Netherlands; Academic Medical Center Amsterdam, University of Amsterdam, Amsterdam, The Netherlands; School of Health Professions Education, University of Maastricht, Maastricht, The Netherlands

**Keywords:** Communication, Faculty development, Teams, Speaking up, Post graduate medical training

## Abstract

The importance of team communication, or more specifically speaking up, for safeguarding quality of patient care is increasingly being endorsed in research findings. However, little is known about speaking up of clinical teachers in postgraduate medical training. In order to determine how clinical teachers demonstrate speaking up in formal teaching team meetings and what factors influence this, the authors carried out an exploratory study based on ethnographic principles. The authors selected 12 teaching teams and observed, audio recorded and analysed the data. Subsequently, during an interview, the program directors reflected on speaking up of those clinical teachers present during the meeting. Finally, the authors analysed iteratively all data, using a template analysis, based on Edmondson’s behaviours of speaking up. The study was conducted from October 2013 to July 2014 and ten teams participated. During the teaching team meetings, the clinical teachers exhibited most of the behaviours of speaking up. “Sharing information” strongly resembles providing information and “talking about mistakes” occurs in a general sense and without commitment of improvement activities. “Asking questions” was often displayed by closed questions and at times several questions simultaneously. The authors identified factors that influence speaking up by clinical teachers: relational, cultural, and professional. The clinical teachers exhibit speaking up, but there is only limited awareness to discuss problems or mistakes and the discussion centred mainly on the question of blame. It is important to take into account the factors that influence speaking up, in order to stimulate open communication during the teaching team meetings.

## Introduction

In order to meet the needs of postgraduate medical training, clinical teachers devote increasing time to teamwork (Eva 2002; Frank et al. 2010; Harden 2011). Teamwork is required to discuss and agree upon a range of issues, including: (1) implementation of the training requirements, (2) supervision of the residents, (3) assessment of the residents’ performance, and (4) safeguarding of patient safety (Edmondson 2003, 2012). Research on the functioning of teams has been carried out by Edmondson, who introduced the term ‘teaming’ in her work on high performance teams (Edmondson 2012). She describes teaming as “a dynamic activity based on members who have teamwork skills and are therefore able to be flexible in working together irrespective of when, where or with whom”. Teaming is characterised by a number of different behaviours, one of which is speaking up. Speaking up is defined as a sincere and direct manner of communication between individuals, including asking questions, seeking feedback and discussing mistakes, and has been shown to have a preventive effect on human error (Edmondson 2012). Not speaking up, for example as a result of anxiety or from a desire to avoid conveying bad news or unwelcome ideas, may protect the individual, but is detrimental for the performance of the team (Edmondson 2003). From the literature it is also known that communication and specific sharing information is essential for learning from mistakes, for both individuals, teams and organizations (Bleakley et al. 2013; Dankoski et al. 2014; Okuyama et al. 2014). We are interested in the way in which clinical teachers apply speaking up behaviours and talk about problems and mistakes. From an earlier study we became aware that the relational communication of clinical teachers is not always their strength and teamwork for postgraduate medical training is not self-evident (Slootweg et al. 2013). What is still unknown is how speaking up is displayed and interpreted by clinical teachers in the context of their discipline-specific meetings and discussions. This qualitative study is intended to make a contribution to understand teamwork among clinical teachers, based on the research question: How do clinical teachers demonstrate the behaviours of speaking up, and what are the factors that influence these behaviours? This knowledge can help with developing interventions to promote speaking up within the teamwork of clinical teachers. We focus particularly on the formal discipline-specific teaching team meetings and how the program directors view the speaking up behaviours, displayed during these meetings. Our aim is to make a contribution to broadening the knowledge about speaking up by healthcare professionals.

## Method

The study was carried out based on a constructivist paradigm. It focuses on participants’ practices in which participants’ accounts are treated as narrative accomplishments rather than as true or false reports of reality (Silverman 2011; Swanwick 2011). As teamwork is complex and calls for solid research, and as much of the existing research on teamwork does not fully reflect this complexity, we combined observation and interviews based on ethnographic principles (Atkinson and Pugsley 2005; Lingard et al. 2012; Silverman 2011; Stewart 2010). Classical ethnography assumes a long-term engagement in a study setting and the collection, through observation and conversational interviews, of data that are analysed to understand the meaning inherent in the everyday activities of a particular group (Bosk 2003). Inspired by this, we opted in this study to observe team communication at a particular point in time, namely during formal teaching team meetings. We subsequently supplemented these observations by conducting interviews with the program director, who were also chairing the teaching team meetings in most cases (Swanwick 2011). The program director has an overview of the collective and has to monitor and promote effective teamwork, which includes encouraging speaking up among clinical teachers.

### Setting and participants

The study was carried out in the Netherlands where residency training programs are coordinated and delivered by university medical centers and in regional affiliated teaching hospitals, where residents work alongside and are supervised by clinical teachers. Program directors have the formal hierarchical position of head of the clinical teaching team, a task for which they are mostly highly motivated (Slootweg et al. 2014). In the Dutch context, it is compulsory that at least four formal meetings on postgraduate medical training are held annually. The meetings are attended by clinical teachers (CT) and the program director (PD); residents may or may not be attending. For this explorative study we used purposeful sampling to select 12 teaching teams with a as diverse as possible range of specialist disciplines, group sizes and hospitals (Pope et al. 2002). The invitations were sent by mail, addressed to the program director. Informed consent was checked at three instances: the program director was asked by email; participants were asked verbally at the start of the meeting; and the program director was asked by means of a form at the start of the interview. The Ethical Review Board of the Academic Medical Center (the university medical center associated with the University of Amsterdam) waived ethical approval for this study.

### Process of data collection and analyses

We started data collection by attending one teaching team meeting for each group and making observations on the basis of the six behaviours of speaking up outlined by Edmondson: (1) asking questions, (2) sharing information, (3) seeking help, (4) experiment with unproven actions, (5) talking about one’s own mistakes and (6) seeking feedback. For each of the meetings, one observer was present (IAS or RvdL) who worked as unobtrusively as possible (Franz 2012). The observations were noted in writing with comments added, and audio recordings were made. The notes and the recordings were coded by two researchers (IAS and RvdL) and were discussed until agreement was reached. The interviews with the program director took place within 1 month after the observations and following the first analysis of the audio fragments from the meeting. We were inspired by the method of Stimulated Recall which means an introspection procedure in which audio-taped fragments of behaviour of speaking up, are replayed by program directors to stimulate recall of the teaching team meeting (Lyle 2003). The interviews were semi-structured and held according to an interview-protocol, based on four analysed audio fragments of speaking up. During the interview the program director was asked, after listening to each fragment, to reflect on the speaking up during the meetings, using a three-point scale: (1) quite limited communication, (2) respectful but guarded communication, and (3) open reciprocal communication (Edmondson 2012). The interviews were all conducted by the first author (IAS) and were recorded.

All the data collected from observations and interviews were iteratively analysed using a template analysis based on the preselected parameters: the six behaviours of speaking up (King et al. 2004). The first step was to analyse the observations, whereby audio fragments were coded using the software package Atlas T. In this process, fragments were selected that were later used for the interviews (Ringsted et al. 2011; Tavakol and Sandars 2014). The choice of fragments was based on a range of different features: all fragments related to team communication and illustrated one of the six behaviours of speaking up. The fragments were on average 3 min long. The second step was to analyse the interview recordings. The sound fragments were also coded using Atlas T, in the template where the data from the observation was analysed. Saturation was reached when all of the data could be analysed using the existing codes and no new codes were identified. See Table 1 for a step-by-step process description.

**Table 1:** Description of the iterative process of analyses: coding, reflection and subsequent outcomes

Coding^a^	Reflection	Outcome of reflection
Teaching team meetings coded (T1, T2, T3)	Discussion RvdL and IAS	Code: add influencing factor to the initial template
Teaching team meetings (T4,T5,T6) and interviews (I1,I2) coded Teaching team meeting 3 double coded by RvdL	Discussion IAS and RvdL, and discussion in research group	The data from the interviews and the observations can be analysed with the same template: (1) influencing factors, (2) reactions, (3) speaking up (SU) *After 6 meetings have been coded, SU gives no new codes*
Interviews (I3,I4,I5) and all date re-coded	Discussion IAS and RvdL	Code: reaction redefined in SU and in the code effect and voice
Teaching team meeting (T7) and interviews (I3,I4,I5) coded	Discussion in research group and with journal club of fellow researchers	Continue coding within the template *After recoding, SU gives no more new codes*
Teaching team meetings (T8,T9,T10) and interviews (I5,I6,I7,I8,I9,I10) coded and interview (I7) double coded by RvdL	Discussion with IAS and RvdL, and discussion in the research group	Code: after 8 interviews, *influencing factors give no more new codes* *Saturation point reached*

^a^I refers to interview numbers and T refers to teaching team numbers

## Results

The data collection and analysis took place in the period from October 2013 to July 2014. Of the 12 teaching teams approached, two did not accept the invitation; one because of the timing of the study and one without indicating the reason: four from non-surgical specialties, four from surgical specialties and two from the supporting disciplines. The teams were from university medical centers (3) and teaching hospitals (7). The team size varied from 7 to 30 clinical teachers. On average there were seven clinical teachers and six residents present during the teaching team meetings (Table 2). The agenda items varied from teaching activities to the assignment of tasks, timetables, legislation, regulations and social activities (Box 1). One of the audio recordings of an observed team meeting failed for technical reasons. The subsequent interview with the program director was therefore conducted on the basis of the notes taken by the observer.

**Table 2:** Observed behaviors of speaking up during the meeting of clinical teachers

Teaching team types	Composition teaching team: clinical teacher/residents	Duration of the meeting	Ease of speaking up according to the program director^a^	Seeking help	Seeking feedback	Talking problems and mistakes	Asking questions	Sharing information	Experiment with unproven actions’
Gastroenterology	8/11	56 min	Guarded	x		x	x	x	
Pediatrics	9/0	37 min	Limited	x	x	x	x	x	
Radiology	8/9	70 min	Limited	x		x	x	x	
Ophthalmology	6/1	77 min	Open	x	x	x	x	x	x
Obstetrics/gynecology	6/7	58 min	Guarded			x	x	x	
Internal medicine	8/0	45 min	Guarded		x	x	x	x	
Plastic and reconstructive Surgery	4/5	50 min	Limited		x	x	x	x	
Obstetrics/gynaecology	4/5	77 min	Guarded	x		x	x	x	
Anesthesiology	9/4	82 min	Open			x	x	x	
Microbiology	14/16	120 min	Limited			x	x	x	

^a^Rating scale: three levels of ease of speaking up, according to the program director

**Box 1:** List of discussion topics

Teaching activity and trajectory
Feedback and portfolio
Faculty development of clinical teachers
Leadership of program director
Introduction program of the residents
Deviding teaching tasks
Timetables of the residents
Quality improvement activity
Students of bachelor and masters medicine
Accreditation and law from national board
Teambuilding and social events

### Speaking up

In answering the first research question, that is how do clinical teachers demonstrate the behaviours of speaking up, we describe successively the six behaviours of speaking up. In Table 2 we illustrated *if* we observed one of the six behaviors per teaching meeting and in the text we focused on *how* the behaviors of speaking up were applied.

The behaviour that clearly manifested itself during the teaching team meetings was “talking about mistakes”, only not one’s own mistakes. During the meeting, mistakes and problems were discussed mainly in a general sense, whereby statements were exchanged and consensus was sought about problems. Conclusions were not drawn or there was evident jumping to conclusions. If clinical teachers talked about undesirable behaviour, it was not always done directly or clearly. Much of the teaching team meeting time was taken up with the behaviour of “sharing information”. Information was given about best practice, previously made agreements and teaching requirements. We observed that during the meeting colleagues regularly shared information with their neighbours in private asides rather than with the team as a whole. Some teaching team meetings consisted wholly of giving information. In these meetings it was the program director who spoke most of the time. The behaviour of “asking questions” was also clearly evident during the meetings. Varying types of questions were asked, both open and—more often—closed. At times several questions were asked simultaneously, and people did not wait for the answer. The “seeking help” behaviour consisted of the program director and clinical teachers asking for assistance in implementing teaching policies, such as to perform teaching tasks, to organize the assessments of the residents and to conduct supervision meetings. This behaviour proceeded relatively automatically and was almost always responded to positively. Program Director (PD) 3 during the interview: “We ask one another for help, including about teaching matters. It’s something we are also used to doing with second opinions in patient care.” The “seeking feedback” behaviour was expressed by asking for feedback about established policy, for example: how do the clinical teachers evaluate the faculty development program of the department. Or seeking feedback about personal performance, for example feedback on the way someone teaches and assesses the residents. In both cases it was the program director who set an example and asked for feedback. No requests for feedback among clinical teachers were observed. The behaviour of “experimenting with unproven actions” was scarcely observed. One of the program directors (PD 4) gave his reaction to this: “We are a cautious team: afraid of new initiatives and particularly good at following rules.”

Summarizing, three of the six behaviors of speaking up were clearly observed during the teaching team meetings. Sharing information primarily takes the form of providing information. Talking about mistakes was mainly expressed in a general sense and does not always lead to a result. Closed questions were often asked, but without waiting for the answer, or no answer came forth. Clinical teachers generally asked one another for assistance. Only the program director asked for feedback, and this only minimally, on issues of policy or about his/her performance as leader of the program. Unproven actions were almost never tried out.

### Influencing factors

The results relating to the second research question address the influencing factors of speaking up by clinical teachers. We identified three factors that influence speaking up mainly through the interviews with the program directors and their reflections on the speaking up audio fragments. (Table 3).

**Table 3:** Influencing factors on speaking up of clinical teachers during teaching team meetings

Relational factors	
Power	PD 1: “sometimes there is a lack of dignity. in the meeting people just want to show who’s the boss”
Leadership	PD 9: what they say is: “medical training is your thing so you should look after that. […..] let’s not spend too much time on it”
Feeling safe	CT 2 during the meeting: “Isn’t this [proposal] rather unexpected?” PD: “No, we are a flexible group.” CT: “Yes, I know that you are flexible, but might it be an idea to brainstorm on this?”
Handling conflict	PD 3 during the meeting when taking his team to task for undesirable behaviour: “Anyone who blushes knows things should be different”
Cultural factors	
History	PD 8: “We have a long history and there’s a lot of going over old ground”
Meeting culture	PD 7: “The core [of the staff] know what we think without having to say it”
Feedback culture	PD 3: “We are all able to say anything to one another: there may be harsh words, but they’re soon followed by a pat on the back”
Professional factors	
Commitment to teaching	PD 6: “This is about teaching, which is something everyone enjoys. Meetings about money are much tougher”
Contribution by residents	PD 8: “The residents don’t say ‘yes’ and that’s the end of it. They really dare to say what they think”
Discipline-specific	PD 9: “It’s typical of anaesthetists to give an OK, shut the door and then do whatever they want”
Personality traits	PD 8: “*He* determines the way *we* work together”

### Relational factors

Speaking up is influenced by whether there is a *power* struggle between the program director, the deputy director and the head of the medical department. Hierarchical relations also played a role here, for example, as one program director indicated (PD 2): “You can’t force people and give them orders”. This power struggle generated an atmosphere, with team members interrupting one another and talking among themselves, causing clinical teachers to drop out. Hardly any questions were asked, there was no drilling down on questions and talking about mistakes mainly centred on the question of blame. The program directors made it clear that they were aware of their *leadership* position as role models for speaking up during the meetings. They indicated that they felt responsible for the teaching and for the well-running of the meetings. They struggled with encouraging others to speak up and seemed mainly to look for ways of sharing information and therefore responsibility. For the program directors it became also clear that *feeling safe* influenced speaking up among clinical teachers. They reflected on examples of questions being asked cautiously, verbosity, talking over one another, contradicting one another and people speaking in defensive, reproachful tones. *Handling conflict* behaviour on the part of the clinical teachers primarily takes the form of safeguarding harmony. The program directors indicated this as a reason during the interviews and stressed the importance of “wanting to be perceived as being nice”. It was noticeable that conflict avoidance behaviour was common in discussing problems without drawing a conclusion, or conversely by jumping to conclusions, ridiculing and not responding to questions.

### Cultural factors

The significance of *past events* was clearly evident during the whole of the data collection. Some teaching teams were typified by the program director reproaches, mutual dissatisfaction and disagreements. PD 2: “Having undergone mediation, our team is damaged. The atmosphere has become embittered”. In these teams, speaking up primarily consisted of laying down information and asking—mainly closed-questions. The converse was also true. In a team that had worked together for a long time, often under the same team leader, the members knew what they could expect of one another, and the program director was able to create an atmosphere of safety. Some teaching team meetings proceeded in a business-like and efficient *meeting culture*. There was an agenda, that seemed to be well-prepared and this resulted in little speaking up during the meeting. There were also teaching team meetings that ran over time, where there was a lack of structure and people frequently talked over one another and failed to listen. Finally, it was apparent that situations that occurred in the department were not always evident in the meetings. In many cases, according to the program director, the real problems were not discussed in the meetings, but in the corridors or in the staff meetings without the residents. Also, it became clear that clinical teachers *handle feedback* differently. There were teams that were characterised by the program director as anxious, where the clinical teachers were not conducive to giving feedback, while there were also teams where giving feedback—even unsolicited feedback—was appropriate for the style of communication. Some teams did little with feedback, giving the desire for harmony as the reason.

### Professional factors

The *teaching* requirements, whether or not bureaucratically imposed, determined the degree to which clinical teachers demonstrated speaking up. There were teaching teams that held meetings because it was required; because the assessment audits required it of them. In the meetings, information was given, not shared, and problems were discussed from the viewpoint of concerns and difficulties. In that meeting fewer clinical teachers were present and the attention of those who were there was frequently elsewhere. According to the program directors, this behaviour was also evident among clinical teachers for whom teaching, and in particular modern teaching programs, had little priority. If *residents* were present at the meetings, they played different roles. They acted as chairman, made proposals, spoke openly about problems, demonstrated strength as a group or as individuals. If the program director had prepared the meeting thoroughly with the residents, there was little speaking up and the meeting was more like an information session. During the interviews the program directors also talked about the *discipline-specific* nature of the specialism and speaking up. The non-surgical specialties showed they were prepared to reflect, while the surgical specialists did not consider themselves meeting types and felt that they were people of few words. The program directors of the supporting specialists suggested that communication was not always seen as a strong point of their specialism.

We observed that every team had at least one conspicuous colleague who influenced with his *personality traits* the speaking up within the team. We heard about the rebel, who was against everything. We heard about the clinical teacher who was known to be an excellent doctor, who spoke reservedly and, if what he was saying was not clear, carried on regardless. We heard about the clinical teachers who did not feel accepted in their position and who expressed their thoughts strongly in the meetings. The program directors spoke during the interviews about the negative attention that these people not only demanded in the group but also received.

## Discussion

This study suggests that in terms of the six behaviours of speaking up teaching teams seem to discuss mistakes and problems, although superficially and almost never personally. These discussions rarely lead to concrete improvement actions. Also, we learn that in the teaching team meetings questions are asked, but that the answers are less important, and that information is given, but that this often meets with little or no response. Edmondson describes the social barriers of teaming and shows that most of the time silence is easier than speaking up (Edmondson 2012). Team members try to minimize interpersonal risk to their image. They avoid speaking up, admitting mistakes, never ask questions or raise tentative ideas, unless they are sure to be right.

The reported findings resonate with the importance of team dynamics in team communication. Liu and Maitlis eleborate in their study on strategic conversation in top team meetings, that communication behaviors, such as decision making and conflict management, are linked with emotions (Liu and Maitlis 2014). The positive emotions—i.e. energetic exchange and amused encounter—draw people together, while the emotional tugs of war—non-empathic interaction, reoccurring confrontation and depleting barrage—drive people apart. The emotional dynamics generated in team meetings increase or diminish the relational distance between people and affect the outcome of the team communication (Liu and Maitlis 2014).

Our findings also resonate with the literature on team communication, reporting that there are “elephants in the room”: issues that seem to be impossible to discuss (Dankoski et al. 2014; Souba et al. 2011). We all see the “elephant”, it gets in our way, but we lack the will, the courage or the skills to discuss problems and mistakes that are quite obvious, or to confront one another with them (Souba et al. 2011). We may therefore not be adequately equipped to resolve mistakes and problems effectively. The price of not speaking up is that the organization does not learn from mistakes (Dankoski et al. 2014; Souba et al. 2011). The fact that in the teaching team meeting the clinical teachers potentially not learn adequately from mistakes may have an impact on the quality of postgraduate medical training. It is likely that clinical teachers could supervise residents better if they themselves knew what it is like to learn from mistakes.

There appear to be three factors that influence speaking up and that can break through or maintain the silence in the teaching team meetings. Clinical teachers are more inclined to avoid conflicts than to discuss them openly. We also see this aspect in the study relating to the question of “What is happening below the surface”(Janss et al. 2012). This study gives an indication that power and conflict have an influence on interpersonal behaviour, with the result that three types of conflicts are inadequately discussed. First there are the task-related conflicts, about people’s ideas on and opinions of tasks. In the teaching team meetings we saw examples of discussing the compulsory teaching tasks and discussions on opinions of modernization of the postgraduate medical training. Differences of opinion among the clinical teachers remained undiscussed: the “elephant” that everyone avoided talking about (Dankoski et al. 2014; Souba et al. 2011). Besides the task-related conflicts, there are also the individual clashes about incompatibilities relating to personal issues. The professional factors, with the personality traits both seem to influence speaking up. Research by Keyton on interaction in dysfunctional teams shows that in every team there are “provokers” and that the team leaders believe that if this provoker is removed or fixed, the team will function more effectively and efficiently (Keyton 1999). What will help here is an open reciprocal communication, with a focus, not on the personality differences, but on the mutual goals (Keyton 1999). The teaching teams, for example, could improve their team communication with a clear and supportive collective ambition on the quality of residency training and the quality of patient care (Slootweg 2015).

Thirdly, Janss et al. indicate that there are process conflicts that refer to logistical or delegational issues, such as responsibilities (Janss et al. 2012). The relational factors and in particular leadership seem to be important in encouraging clinical teachers to take responsibility for teaching and to practice speaking up (Dankoski et al. 2014). From the study on the willingness in surgeon behaviour to speak up about training, it is known that the senior surgeon does indeed have an important role in improving the communication between junior and senior clinicians to enhance patient safety (Barzallo Salazar et al. 2014).

The cultural factor seems to add significance to the results of speaking up. Here factors are ignored that have played a role for some time, as deep-rooted values, norms and habits. One example is experimenting with new behaviour that scarcely features at all. Edmondson shows how important an atmosphere of safety is for the learning process of collective discussions and experimenting with new behaviours. Safety, as we indicated earlier, is not always felt in teaching teams (Edmondson 1999, 2003, 2012).

The strength of this study is in the use of both observations and interviews. The data from the interviews has reinforced the data from the observations and added to its significance. We analysed the audio fragments rather than taking the spoken text verbatim, which generated a thorough analysis. The six behaviours of speaking up give a clear focus to the data and make an accurate contribution to providing an answer to the research questions. The limitation of this study is in the assumption that one meeting is representative for the team communication among clinical teachers. For this reason, future research should focus on the team communication on postgraduate medical training in daily practice. A further limitation is the fact that this study was carried out in the Netherlands where a culture of open feedback may be more accepted than in other cultures. International comparative research in the area of speaking up would be interesting.

## Practical implication and future research

The results of this study can be used as a source of inspiration for program directors to improve speaking up as a shared learning process that could help clinical teachers in their collaborative task of training residents. Based on this study we feel the following recommendations could facilitate speaking up behaviours in teaching teams:

- Introduce the observation of the teaching team meetings as a learning intervention, including the follow up discussion with the program director of the strengths and improvement points;
- Sharing knowledge on the importance of efficient meetings for team communication amongst clinical teachers;
- Support clinical teachers to demonstrate speaking up behavior during the teaching meetings and also during other more patient-oriented meetings;
- Show leadership by the program director in stimulating clinical teachers in this shared learning process.

It is our expectation that when clinical teachers demonstrate speaking up behaviors during the teaching meetings, the open and transparent communication of safeguarding and improving the quality of postgraduate medical teaching may increase. This can have a positive impact on the quality of the teaching and assessment of the residents (Slootweg 2015).

Follow-up research should focus on a more longitudinal study and more thoroughly observing team communication among clinical teachers to investigate which influencing factor is most defining for whom and for which specialty. Further, now that we know more about the formal teaching team meeting, it would be interesting to analyse the way that mistakes in postgraduate medical training are discussed by clinical teachers in an informal setting.

## Conclusion

Clinical teachers demonstrate behaviours of speaking up during teaching team meetings, whereby it appears that the problematic topics are only discussed to a limited degree. Mistakes and conflicts are mainly discussed in a general sense, and are often neither directed at the individual nor adequately result-oriented, which means that the positive effect that speaking up could have on the quality of postgraduate medical training is largely lacking. If clinical teachers are to develop the behaviours of speaking up, it is important to take into account the relational, cultural and professional factors that influence speaking up in order to stimulate sincere and direct communication during the teaching team meetings.
